# Emerging key roles for P2X receptors in the kidney

**DOI:** 10.3389/fphys.2013.00262

**Published:** 2013-09-27

**Authors:** R. E. Birch, E. M. Schwiebert, C. M. Peppiatt-Wildman, S. S. Wildman

**Affiliations:** ^1^Medway School of Pharmacy, The Universities of Kent and GreenwichKent, UK; ^2^Discovery BioMed Inc. Birmingham, AL, USA

**Keywords:** P2X receptor, P2 receptors, kidney, renal circulation, renal tubular transport, inborn errors, P2X7 receptor, pathology

## Abstract

P2X ionotropic non-selective cation channels are expressed throughout the kidney and are activated in a paracrine or autocrine manner following the binding of extracellular ATP and related extracellular nucleotides. Whilst there is a wealth of literature describing a regulatory role of P2 receptors (P2R) in the kidney, there are significantly less data on the regulatory role of P2X receptors (P2XR) compared with that described for metabotropic P2Y. Much of the historical literature describing a role for P2XR in the kidney has focused heavily on the role of P2X1R in the autoregulation of renal blood flow. More recently, however, there has been a plethora of manuscripts providing compelling evidence for additional roles for P2XR in both kidney health and disease. This review summarizes the current evidence for the involvement of P2XR in the regulation of renal tubular and vascular function, and highlights the novel data describing their putative roles in regulating physiological and pathophysiological processes in the kidney.

## Introduction

Extracellular ATP and related nucleotides have been shown to contribute to complex autocrine and/or paracrine signaling networks throughout the body following their activation of P2 receptors (P2R; formerly termed purinoceptor; Burnstock and Knight, 2004). The P2 family of receptors is divided into metabotropic G protein-coupled P2Y receptors (P2YR) and ionotropic ligand-gated P2X receptors (P2XR), which act as non-selective cation channels (Fredholm et al., 1994). There are eight pharmacologically distinct P2YR (P2Y_1, 2, 4, 6, 11−14_) and seven unique P2XR subunits (P2X1-7), which can form seven homomeric assemblies, seven established heteromeric assemblies (P2X1/2, 1/4, 1/5, 2/3, 2/6, 4/6, 4/7), as well as several predicted heteromeric assemblies. Heterologous expression systems have been used to demonstrate that P2XR can readily form functional trimeric or hexameric assemblies (North, 2002). The actual number of subunits present in native cell multimeric assemblies is still debated, however, the trimer is favored (North, 2002). It is established that there are an abundance of P2R in a variety of cell types throughout the body, linked to numerous physiological processes such as regulation of other membrane-located ion channels, cell-to-cell communication, secretion of cytokines, metabolic processes such as glugoneogenesis, cell proliferation, and cell death (Burnstock and Knight, 2004).

**Figure 1:**
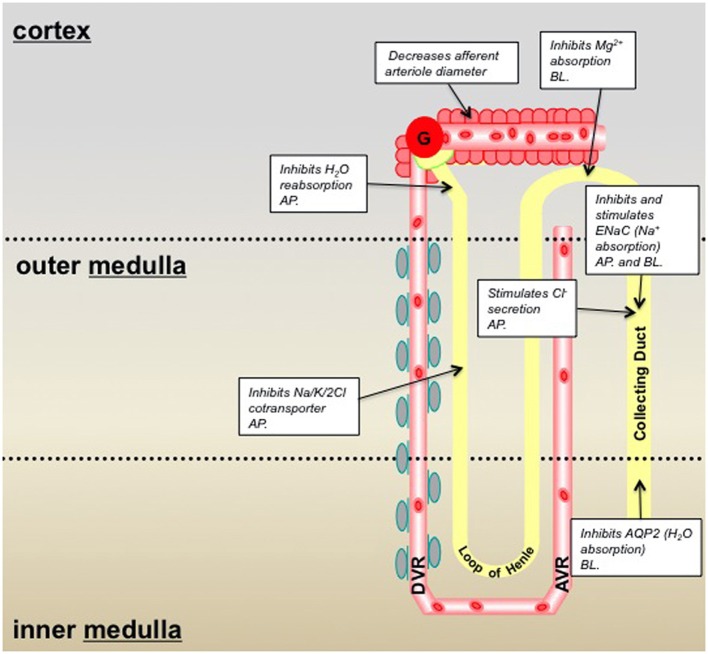
**Summary of P2X receptor-mediated effects in the kidney and epithelial cell lines derived from specific nephron segments; pre-2010/11.** Information taken from: Wildman and King, 2008; Bailey et al., 2012. Key: *AP*, denotes that the P2X receptors are localized on the epithelial cell *apical* membrane; *BL*, denotes localization on the *basolateral* membrane.

**Figure 2:**
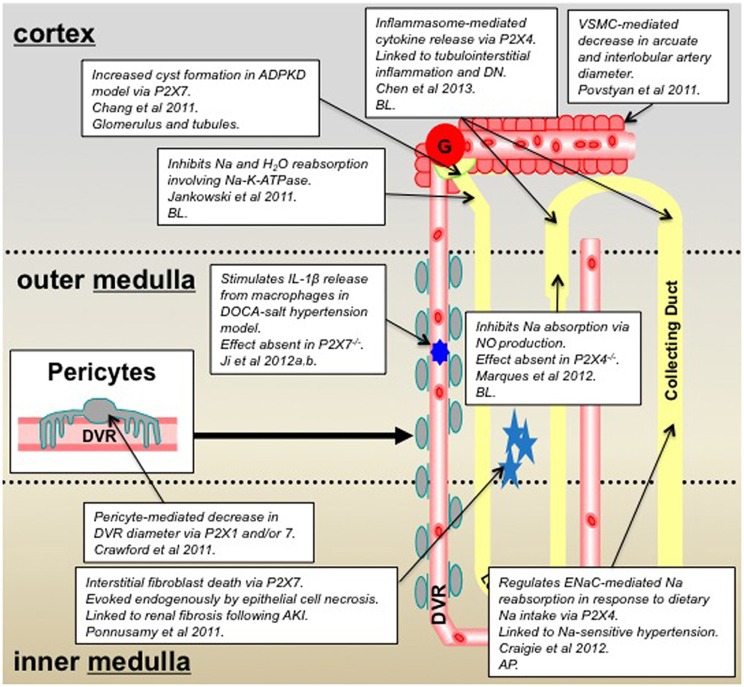
**Summary of P2X receptor-mediated effects in the kidney and epithelial cell lines derived from specific nephron segments; 2011–2013 inclusive.** Key: *AP*, denotes that the P2X receptors are localized on the epithelial cell *apical* membrane; *BL*, denotes localization on the *basolateral* membrane; ADPKD, autosomal dominant polycystic kidney disease; DN, diabetic nephropathy; VSMC, vascular smooth muscle cell; DVR, descending vasa recta.

It is perhaps not surprising that P2R are ubiquitously expressed throughout the kidney, in both cortical and medullary vascular and tubular compartments. They contribute to a diverse range of physiological and pathophysiological processes and yet the localization of specific P2R subtypes can be highly regional, overlapping, and often debated. Much of the renal literature published to date focuses on the role of metabotropic P2YR, and in particular in their regulation of sodium (Na) and water transport in the collecting duct (CD; Kishore et al., 2009; Wildman et al., 2009; Vallon and Rieg, 2011), with P2XR being somewhat overlooked (with the exception of their role in regulating afferent arteriole diameter; see summary Figure 1). Arguably a dogma exists that P2XR play little or no role in kidney function. However, a recent glut of manuscripts provides compelling evidence for P2XR playing key roles in the kidney. This review will discuss the most recent advances in the field of “renal P2XR” [i.e., the key literature of the last 3-years (2010–2013)], focusing on the tubular and vascular localization of P2XR and their function(s) in both the physiological and pathological setting (see summary Figure 2).

## New roles for P2XR in renal tubular transport

Expression of P2XR varies throughout the nephron. P2X4R and P2X6R are expressed in the proximal tubule, distal tubule, loop of Henle and CD, making these receptor subtypes the most widely distributed (Unwin et al., 2003). P2X1R and P2X7R are localized predominantly in Bowman's capsule and the CD and are more widely distributed in vascular networks (as discussed in the next section; Inscho et al., 2003; Vitzthum et al., 2004; Osmond and Inscho, 2010; Crawford et al., 2011). Immunohistochemical studies have also demonstrated low levels of expression for P2X2R and P2X5R in the cortical and medullary CDs (Turner et al., 2003; Wildman et al., 2008). The putative roles previously described for P2XR in the nephron have included inhibition of fluid reabsorption in the proximal tubule, inhibition of magnesium absorption in the distal tubule, and inhibition of AQP2-mediated water absorption and modulation (inhibiting and potentiating) of ENaC-mediated Na^+^ absorption/reabsorption in the CD (Bailey et al., 2012; see summary Figure 1).

Novel data obtained from pharmacological experiments that utilized P2XR selective agonists, complimented by studies in knockout mice (^−/−^), now provide compelling evidence for a functional role for P2XR in the medullary thick ascending limb (mTAL) (Marques et al., 2012). It is well-established that NaCl enters cells of the TAL via the apical Na/H exchanger and Na-K-2Cl co-transporter and leaves the cell via basolateral Na-K-ATPase, and nitric oxide (NO) inhibits both the Na/H exchanger and Na-K-ATPase to regulate ion transport. Experiments to determine the effect of basolateral ATP on NaCl absorption in isolated perfused mouse mTALs, using the electrical measurement of equivalent short-circuit current, demonstrated that basolateral ATP attenuated the absorptive short-circuit current (Marques et al., 2012). Authors used P2XR selective agonists and antagonists to demonstrate the effect was mediated, not via P2YR as might have been expected, but via P2XR. Experiments reproduced in both P2X7R^−/−^ and P2X4R^−/−^ concluded that the ATP-inhibition of transport was reduced in the P2X4R^−/−^ animals thus indicating a key role for P2X4R. This finding was further corroborated by PCR experiments, which confirmed the presence of P2X4R mRNA, together with P2X1R and P2X5R mRNA in isolated mTAL (Marques et al., 2012). In addition, complimentary studies seeking to determine the factors responsible for flow-mediated NO production in the mTAL identified ATP as a candidate. Researchers used P2YR and P2XR selective antagonists to demonstrate a role for basolateral P2XR (and apical P2YR) in ATP-mediated, flow-induced production of NO in the mTAL (Cabral et al., 2012). Collectively, data from these studies suggest that ATP, released by increased tubular flow rate, acts on basolaterally-expressed P2X4R (potentially a heteromeric assembly, with either P2X1R or P2X5R), to increase NO production, which ultimately inhibits NaCl reabsorption in the mTAL. However, the mechanism by which luminal ATP activates basolaterally-expressed P2X4 receptors is yet to be elucidated.

Studies performed in our laboratory and with collaborators have similarly identified roles for P2X4R, and potentially P2X7R, in the regulation of Na reabsorption. However, our studies have focused on the CD rather than the TAL. Initially using M1 cells, an immortalized mouse cortical CD cell line, in combination with electrophysiology techniques we identified ionotropic P2XR-mediated channel activity (Birch et al., 2013a). Application of ATP to outside-out patches activated single-channel openings, from multiple receptor subtypes, with single-channel properties similar to those of P2XR previously identified in other cell types (Evans, 1996; Birch et al., 2013a). Characterization of the exact P2XR subtypes mediating the single-channel activity is a current focus of the group. Initial observations in rat CD principal cells *in vitro* demonstrated that apical P2X4R modulates ENaC (epithelial Na channel) activity: high concentrations of luminal ATP evoked P2X4R-mediated inhibition of ENaC activity, whereas low concentrations conversely potentiated ENaC activity (Wildman et al., 2008). In addition to our *in vitro* functional data we have used P2X4R^−/−^ mice to demonstrate an *in vivo* role for P2X4R in Na reabsorption and salt sensitivity (Craigie et al., 2012). Data from our most recent *in vivo* renal clearance studies have revealed a raised mean arterial blood pressure (MABP) in P2X4R^−/−^ when compared with wild-type (WT) littermates. When dietary Na was altered to what is termed a low Na diet (0.03% compared to 0.3% in a standard diet), the reduction in dietary Na significantly reduced MABP in the P2X4R^−/−^ but not in WT. Interestingly, when comparing the ENaC-mediated reabsorption for WT and P2X4R^−/−^ on a standard diet there was no significant difference. However, when on a low Na diet the P2X4R^−/−^ mice exhibited a significantly reduced fractional Na excretion compared with WT, indicating this group of animals were unable to increase ENaC-mediated Na reabsorption in the absence of P2X4R. These data collectively suggest that P2X4R^−/−^ mice have salt-sensitive blood pressure and are unable to increase their ENaC-mediated sodium reabsorption when dietary sodium is low. The latter finding is compatible with our previous *in vitro* data that describes a P2X4-mediated potentiation of ENaC activity when extracellular sodium is low. Thus, P2X4 may act as an intrinsic regulator of ENaC activity in the aldosterone-sensitive distal nephron to moderate Na reabsorption in response to changes is Na load. Perhaps of note, *in vivo* pharmacological studies performed in rats, whereby P2XR agonists were intravenously infused showed a marked, progressively-increasing diuresis, together with an increase in the Na excretion rate. Authors concluded that activation of P2XR increased renal Na and water excretion in rats via a Na-K-ATPase-dependent mechanism (Jankowski et al., 2011). Evidently, P2X4R in the CD may feature in the complexity of essential hypertension.

Whilst the precise mechanism(s) responsible for P2XR-mediated increases in Na excretion require clarification, the evidence in favor of significant functional roles for P2XR in the nephron is increasing. This is further confirmed by pathophysiological findings that provide evidence for the involvement of P2XR in kidney dysfunction. P2X4R have been highlighted as key receptors in inflammasome activation and have been linked to diabetic nephropathy (DN) (Chen et al., 2013). It is well-established that tubulointerstitial inflammation is involved in the development of DN, and is mediated by key inflammatory cytokines. Chen and colleagues demonstrated that extracellular ATP causes P2X4R to activate the NOD-like receptor 3 (NLRP3) inflammasome, causing cytokine maturation and release (Chen et al., 2013). In light of the potential link between P2X4R, inflammasome activation, and DN, P2X4R signaling in NLRP3 inflammasomes was investigated using renal biopsies from patients with type-2 diabetes. Authors reported increased expression of P2X4R in renal tubular epithelial cells of patients with type-2 diabetes compared with non-diabetic patients, and that P2X4R expression correlated with expression of IL-1 and IL-18 (Chen et al., 2013). This finding is perhaps not surprising since ATP is one of the best established damage-associated molecular patterns (DAMPs), and as such plays an important role in inflammation and immunity by functioning as a signal of cell damage, stress or death during injury and disease (Chen and Nunez, 2010). A cautionary note: whilst these studies substantiate the hypothesis that P2X4R blockade would be a successful therapeutic strategy in attenuating DN, there is evidence to suggest this may lead to hypertension by disrupting sodium transport in the distal nephron (as previously discussed). This highlights the fact that although P2XR have emerged as attractive therapeutic targets for many pathophysiological conditions, their widespread expression and diverse roles means the manipulation of these receptors is extremely complex.

Activation of P2X7R (albeit via a more established mechanism), like P2X4R, induces downstream inflammatory events, including the NALP3 inflammasome/caspase-1-dependent maturation of IL-1β and IL-18, and their subsequent release from various myeloid cell types (Idzko et al., 2007). There is now also evidence for Madin-Darby canine kidney (MDCK) epithelial cells expressing functional P2X7R, as well as TLR4 and molecules associated with the NALP3 inflammasome (Jalilian et al., 2012), perhaps highlighting their importance in *renal* inflammatory injury and *renal* disease. P2X7R are perhaps the P2XR subtype most linked to pathophysiology of the kidney. Historically, P2X7R (which can form pores under certain circumstances) are known to be pro-inflammatory, and whilst they are extensively expressed in cells of the immune system they are not *highly* expressed in healthy kidney tissue (Hillman et al., 2005). Recent additional lines of evidence that describe alternative roles for P2X7R in kidney dysfunction have revealed a role for P2X7R in: the disrupted calcium homeostasis in peripheral blood mononuclear cells of chronic kidney disease patients (Lajdova et al., 2012), P2XR-mediated cytogenesis in polycystic kidney disease (Chang et al., 2011), interstitial fibroblast cell death following ATP release from necrotic renal epithelial cells (Ponnusamy et al., 2011), and a crucial role in driving hypertension and renal injury (Ji et al., 2012b). It seems that both P2X4R and P2X7R are evermore commonly associated with kidney dysfunction. With this in mind we have utilized P2X4R^−/−^ and P2X7R^−/−^ mouse models and noted that in the P2X4R^−/−^, mRNA for P2X7R was reduced and vice versa, which collectively indicates an interaction of P2X4R with P2X7R at the expressional level to (patho)physiologically regulate renal function (Birch et al., 2013b; Craigie et al., 2013). The specific role of P2XR in renal pathophysiology will be discussed below in greater detail.

## New roles for P2XR and renal vasculature

In addition to tubular epithelial cell expression, P2R are expressed in renal vascular and glomerular cells of the medulla and cortex (Bailey et al., 2012). The role of P2R in renal vasculature is complex and dependent on many factors including species, vascular tone, and variable P2R expression patterns. Despite this, numerous functional studies have previously established that P2R have a regulatory role in the renal vasculature (Inscho et al., 1992; Weihprecht et al., 1992; Eltze and Ullrich, 1996; Inscho, 2001; Rost et al., 2002; Eppel et al., 2006a). Much of the historical evidence surrounding a role for P2XR in the regulation of renal blood flow focuses on the P2X1R, most likely a reflection of this being the key receptor expressed by vascular smooth muscle cells (VSMCs). P2X1R are expressed in afferent arterioles but not in efferent arterioles (Osmond and Inscho, 2010) and functional data suggest that ATP-mediated activation of P2X1R represents an important mechanism for regulating renal blood flow and glomerular capillary pressure by regulating afferent arteriolar resistance and in pressure-dependent autoregulatory adjustments in afferent arteriolar diameter (Inscho et al., 2003). *In vivo* studies in which rabbits were infused intrarenally with α, β-methylene ATP described a reduction in both cortical and medullary blood flow that implicated P2XR in the regulation of regional renal blood flow (Eppel et al., 2006b). The interpretation of these former studies have, however, been challenged by recent mouse micropuncture experiments, that have raised questions as to the role of P2XR specifically in tubuloglomerular feedback (TGF), which is the mechanism responsible for matching glomerular filtration to perfusion in the loop of Henle (Schnermann, 2011). The author of this study reported that both intravenous infusion and luminal perfusion of inhibitors of P2R (at a dose that significantly reduced the blood pressure response to α, β-methylene ATP), failed to affect the TGF response to saturating increases in loop perfusion rate. Moreover, macula densa release of nucleotides appeared not to be involved in the vasoconstriction that accompanies increased loop flow *in vivo* (Schnermann, 2011). These findings are in contrast to an *in vivo* study in which anaesthetized rats were treated with Ip_5_I, a potent P2X1R antagonist, and showed a significant loss of autoregulatory ability, whereas in anaesthetized rats treated with DPCPX, a potent A1R antagonist, autoregulatory control of renal blood flow remained intact (Osmond and Inscho, 2010). The disparity noted above may reflect the acknowledged differences in the relative expression of P2XR between species. Collectively these data suggest that P2X1R are implicated in renal autoregulatory (and maybe not TGF) responses, although the relative contribution of P2XR to these complex physiological processes requires further clarification and may be species dependent. Furthermore, it is possible that ATP and adenosine signaling pathways integrate to mediate TGF. ATP released in response to increased perfusion pressure and/or increases in tubular concentrations could mediate vasoconstriction of the afferent arteriole via P2XR, but might also be dephosphorylated to form adenosine, which may compound the afferent arteriole vasoconstriction.

In a recent study utilizing the live kidney slice model to investigate regulation of medullary blood flow (MBF) we reported a role for P2XR (and P2YR) in the regulation of vasa recta diameter via contractile pericytes (Crawford et al., 2011). Data described an ATP-mediated constriction of *in situ* vasa recta via contractile pericytes and also demonstrated attenuation of the ATP-mediated constriction in the presence of the P2XR antagonist PPADS. Additionally pericytes constricted vasa recta diameter when live slices were superfused with 2meSATP and BzATP, which act at P2XR. PCR experiments performed on isolated vasa recta to investigate P2R mRNA expression combined with the pharmacological data acquired in functional experiments indicated a putative role for both P2X1R and P2X7R in the regulation of MBF (Crawford et al., 2011). Since pericytes are considered to be relatively undifferentiated having the ability to differentiate further in to VSMCs, it is interesting to note that calcium entry via P2XR has also recently been implicated in the contractile mechanism of renal VSMCs isolated from arcuate arteries and interlobular arteries (Povstyan et al., 2011), thus providing additional evidence for P2XR having a functional role in mediating VSMC contraction in other vascular cells of the kidney. Regarding the functional role of P2XR in pericytes *per se*, P2XR (particularly P2X7R) have also been implicated in the regulation of the retinal pericytes (Sugiyama et al., 2004, 2005) and in the regulation of pericytes of spiral ligament capillaries in the cochlea (Wu et al., 2011).

Given the established role for P2XR in the regulation of vascular cells throughout the body it is not surprising that there are emergent studies in the renal field also highlighting their significance in renal blood flow regulation. What is striking, however, is the accumulating evidence that points toward P2XR playing a critical role in renal pathophysiology, whether the etiology is vascular or tubular in origin.

## P2XR and renal pathophysiology

With current funding bodies placing ever increasing importance on translational “bench to bedside” research it is particularly exciting when a molecule or group of molecules emerge as having prominent roles in organ pathology. As eluded to already in this article, there is a compelling mass of evidence that indicates P2XR play crucial and divergent roles in renal pathophysiology. As such, P2XR now represent novel putative targets for renal disease intervention and treatment strategies (North and Jarvis, 2013), and are currently the subject of avid investigation by multiple major international pharmaceutical companies.

Much of the current focus on the potential role of P2XR in renal disease is focused on the homomeric P2X7R, this ligand-gated cation channel being unique in terms of both its structure and function. Unlike its other family members it is thought to exist only as a homomer and has an extended C-terminus with 200 extra amino acid residues, which is thought to be pivotal in regulating its function, being involved in: determining cellular localization, stimulation of various signaling cascades, protein-protein interactions, and post-translational modification of the receptor itself (Costa-Junior et al., 2011). Sustained stimulation of P2X7R with high agonist concentrations (mM) is classically associated with the formation of large transmembrane pores that disrupt the ion gradients within cells resulting in subsequent cell death by either apoptosis or necrosis (Surprenant et al., 1996). The P2X7R is typically expressed by immune cells and mediates release of pro-inflammatory cytokines, prominently interleukin-1 beta (IL-1β), via activation of caspase 1 (Ferrari et al., 1997; Costa-Junior et al., 2011). In contrast to the healthy kidney in which P2X7R is virtually undetected, P2X7R expression is readily detected in inflammatory renal diseases, such as glomerulonephritis (Vonend et al., 2004). The role of P2X7R in renal pathophysiology is not however limited to inflammatory disease. Indeed current topical studies demonstrate that P2X7R plays a detrimental role in a broad range of renal pathologies that will be discussed below.

A recent study has suggested that the pro-inflammatory nature of P2X7R could contribute to the development of hypertension and consequential renal injury. The functional role of P2X7R in hypertension was investigated using WT and P2X7^−/−^ mice rendered hypertensive by a high salt diet and deoxycorticosterone acetate (DOCA) treatment (Ji et al., 2012b). After DOCA-salt treatment, P2X7R mRNA and protein expression was increased in WT mice, accompanied by a greater increase in systolic blood pressure (SBP), significantly greater than that of P2X7^−/−^ mice. Inflammatory cell infiltration was reduced in P2X7^−/−^ mice, and IL-1β release from macrophages was inhibited. Furthermore, the downstream effectors of IL-1β signaling, COX-2 and ROS were also affected; COX-2 expression being reduced in P2X7^−/−^ mice and anti-oxidant levels being increased in their serum. Authors of the study suggested that blockade of the P2X7R-mediated inflammatory responses protect renal function against P2X7R-associated renal injury, this being reflected by reduced albuminuria and renal interstitial fibrosis, and increased creatinine clearance (Ji et al., 2012b). With regard to hypertension, both WT and P2X7^−/−^ exhibited initial increases in SBP following DOCA-salt treatment, and only at day-4 of treatment were differences in SBP observed between the two animal groups; P2X7^−/−^ mice having significantly lower SBP than WT mice. This study built on previous observations demonstrating that immune cell infiltration was pivotal to the development of salt-sensitive hypertension (Franco et al., 2007), authors of this preceding study reported P2X7R expression by immune cells exacerbated renal injury by instigating a vicious cycle of inflammation. Additional *in vivo* evidence to support this has been attained from pharmacological studies performed in Dahl salt-sensitive (DS) rats (Ji et al., 2012a). Hypertensive rats were infused with two P2X7R selective antagonists (brilliant blue G and A-438079); the salt-sensitive hypertension, urinary protein or albumin excretion, renal interstitial fibrosis, and macrophage and T-cell infiltration in the DS rats was markedly reduced, whilst creatinine clearance significantly improved (Ji et al., 2012a). Collectively these novel studies provide persuasive evidence that the P2X7R is critical in hypertension and renal injury progression, thus highlighting the therapeutic potential for this receptor in a number of disease states underlying salt-sensitive hypertension, including but not limited to, hyperaldosteronism.

As previously mentioned, blockade of P2X7R has been shown to inhibit IL-1β release from renal macrophages (Ji et al., 2012b), and this may have significant consequences for the underlying mechanisms of certain forms of renal disease. Renal fibroblasts are activated by IL-1β amongst other cytokines and due to the P2X7R/IL-1β axis it seems pertinent to assume P2X7R-mediated attenuation of fibroblast activation could occur. Whilst this may have beneficial effects in terms of reducing renal interstitial fibrosis, activation of renal fibroblasts post acute kidney injury (AKI) is vital for normal renal repair. AKI is typically characterized by renal tubular cell death and associated release of high concentrations of ATP into the interstitium, combined with the associated damage to the tubular basement membrane, to which interstitial fibroblasts are directly connected; this milieu provides an ideal setting for P2X7R to play a prominent role in either AKI associated fibrosis or renal repair. To investigate this paradigm, a recent study investigated the effects of supernatant from necrotic renal proximal tubular cells (RPTC) on the viability of renal fibroblasts (Ponnusamy et al., 2011). The supernatant from necrotic RPTC was shown to induce the expression of P2X7R in cultured rat renal interstitial fibroblast cells (NRK-49F), and the ATP released from necrotic RPTC induced NRK-49F cell death by a P2X7R-mediated mechanism, therefore demonstrating a deleterious direct renal epithelial-fibroblast cross-talk pathway (Ponnusamy et al., 2011). It was concluded that damaged renal epithelial cells could directly induce the death of renal interstitial fibroblasts by ATP activation of P2X7R. This evidence was not, however, in accordance with a previous study which showed that the number of Epo-TAg (a genetic marker for cortical interstitial fibroblasts) expressing fibroblasts is reduced in the injured kidney, but upon intense stimulation such as severe anemia or hypoxia, there was a greater increase in Epo-TAg expressing fibroblasts in the injured kidney which consequently narrowed the disproportion between the injured and uninjured kidney. Authors conclude that rather than fibroblasts being destroyed in the event of AKI, the threshold for stimulated gene expression is increased (Maxwell et al., 1997), which could possibly be mediated by P2X7R. So it is unclear if AKI results in a reduced number of renal interstitial fibroblasts or an alteration in cell properties.

Renal interstitial fibroblasts have both beneficial and deleterious effects on the kidney by playing a key role in renal repair and renal fibrosis, respectively. Consequently, interstitial fibroblast cell proliferation, activation, and death are tightly regulated by numerous factors. It is certainly plausible that ATP is one such factor and P2X7R is an important mediator. It has been shown *in vitro* that P2X7R activation directly influences interstitial fibroblast cell death (Ponnusamy et al., 2011). However, the possibility exists that P2X7R also indirectly influences fibroblast activation by inducing IL-1β release from infiltrating immune cells (Ji et al., 2012b). Both studies propose P2X7R as a therapeutic target. Although pharmacological blockade of P2X7R was highly effective at attenuating renal fibrosis, the implications this may have on the repair response post-AKI need to be considered.

Lastly, in addition to the pro-inflammatory and pro-death functions of P2X7R discussed in preceding paragraphs there seems also to be a role for P2X7R in autosomal dominant polycystic kidney disease (ADPKD). This common, yet complex, genetic renal disease occurs in response to mutations in either the PKD1 or PKD2 gene (Mochizuki et al., 2013), and is characterized by progressive cyst enlargement due to excess fluid secretion and proliferation of renal tubular epithelial cells (Torres et al., 2009). Whilst this condition is a major cause of end stage renal disease, there still lacks a clinically approved therapy, primarily due to the slow progression of the disease and complexity of its development. The current focus is to assess the potential of combination therapies targeting either cyst development through inhibition of cell proliferation, and/or cyst growth through attenuation of fluid secretion. Recent data yielded from the zebrafish model of ADPKD where PKD2 is knocked-down, described increased P2X7R mRNA expression at an early stage, which coincided with cyst formation in the glomerulus and tubular regions in 75% of PKD2 morphants. Pharmacological blockade with a P2X7R antagonist OxATP reduced the frequency of cyst formation to 35%, suggesting that P2X7R contributes in some way to cyst formation in ADPKD (Chang et al., 2011). PKD2 morphants demonstrate increased ERK activation in primary cilia, which is linked to enhanced cell proliferation and fluid secretion and hence cyst enlargement in PKD. Previous studies have demonstrated that P2X7R acts as a flow sensor for ERK activation in osteoblasts (Okumura et al., 2008). Given that authors describe inhibition of P2X7R as suppressing the elevated ERK activation in PKD2 morphants by over 50%, coupled with the fact cilium bending has been shown to trigger ATP release, suggests a similar P2X7R-coupled mechanism may be present in renal epithelial cells. This hypothesis is further substantiated by findings that demonstrated inhibition of P2X7R did not suppress cyst progression through the traditional anti-inflammatory mechanism (Chang et al., 2011; Praetorius and Leipziger, 2013). Whilst extremely novel, these data are, however, at odds with previously published data collected from studies using the MDCK model of cyst formation. Here authors agreed that ERK activation is an important factor in cyst growth. However, they found no evidence for the involvement of a P2XR, and concluded that a P2YR is responsible for cAMP-dependent activation of the ERK pathway and cyst growth (Turner et al., 2007).

## Summary

P2XR represent a receptor group important in the regulation of tubular and vascular function in both kidney health and disease (see Figures 1, 2 for a summary). Much of the recent data providing compelling functional evidence that substantiate this phenomenon has been generated from knockout mouse studies, which further attests to the importance of these models in improving our knowledge of how kidney function is regulated. As a result of these studies there appear to be three key P2XR subunits/subtypes, P2X1, 4, and 7, which have emerged as having numerous roles in the regulation of kidney function. As such, these receptor subtypes not only represent favorable novel therapeutic targets in multiple pathological settings, but also represent a novel focus for future research on the role of nucleotides in the kidney.

### Conflict of interest statement

The authors declare that the research was conducted in the absence of any commercial or financial relationships that could be construed as a potential conflict of interest.
